# Supplementary material for: A high-resolution mRNA expression time course of embryonic development in zebrafish
Source: eLife. 2017 Nov 16;6:e30860. doi: 10.7554/eLife.30860 (PMC5690287; doi:10.7554/eLife.30860)
Supplement: Supplementary file 6. [file elife-30860-supp6.zip › biolayout-clusters-files/Cluster135-genes.html]

Cluster135


# Cluster135: Genes

| Ensembl ID | Gene Name | Chr | Start | End | Biotype |
| --- | --- | --- | --- | --- | --- |
| ENSDARG00000092436 | BX640584.1 | 4 | 50480544 | 50486215 | protein\_coding |
| ENSDARG00000090400 | CR388363.1 | 4 | 58803634 | 58827891 | protein\_coding |
| ENSDARG00000099043 | CR847895.1 | 4 | 29391015 | 29405633 | protein\_coding |
| ENSDARG00000099760 | CT573263.1 | 4 | 64866060 | 64872970 | protein\_coding |
| ENSDARG00000036774 | rgs12b | 1 | 40498799 | 40587799 | protein\_coding |
| ENSDARG00000088214 | si:dkey-23a23.3 | 4 | 29008131 | 29021917 | protein\_coding |
| ENSDARG00000098796 | znf1122 | 4 | 45818007 | 45994340 | protein\_coding |
| ENSDARG00000093424 | znf1135 | 4 | 48478499 | 48486431 | protein\_coding |
| ENSDARG00000102735 | znf1141 | 4 | 49306725 | 49428852 | protein\_coding |
| ENSDARG00000100512 | znf986 | 4 | 54145610 | 54153623 | protein\_coding |
